# Astronaut omics and the impact of space on the human body at scale

**DOI:** 10.1038/s41467-024-47237-0

**Published:** 2024-06-11

**Authors:** Lindsay A. Rutter, Henry Cope, Matthew J. MacKay, Raúl Herranz, Saswati Das, Sergey A. Ponomarev, Sylvain V. Costes, Amber M. Paul, Richard Barker, Deanne M. Taylor, Daniela Bezdan, Nathaniel J. Szewczyk, Masafumi Muratani, Christopher E. Mason, Stefania Giacomello

**Affiliations:** 1 Transborder Medical Research Center, University of Tsukuba, 305-8575 Tsukuba, Japan; 2 Department of Genome Biology, Institute of Medicine, University of Tsukuba, 305-8575 Tsukuba, Japan; 3 School of Medicine, University of Nottingham, Derby, DE22 3DT UK; 4 Department of Physiology and Biophysics, Weill Cornell Medicine, New York, NY 10065 USA; 5 The HRH Prince Alwaleed Bin Talal Bin Abdulaziz Alsaud Institute for Computational Biomedicine, Weill Cornell Medicine, New York, NY 10021 USA; 6 The WorldQuant Initiative for Quantitative Prediction, Weill Cornell Medicine, New York, NY 10065 USA; 7 Centro de Investigaciones Biológicas “Margarita Salas” (CSIC), Ramiro de Maeztu 9, Madrid, 28040 Spain; 8 Department of Biochemistry, Atal Bihari Vajpayee Institute of Medical Sciences & Dr. Ram Manohar Lohia Hospital, New Delhi, 110001 India; 9 Department of Immunology and Microbiology, Institute for the Biomedical Problems, Russian Academy of Sciences, 123007 Moscow, Russia; 10 Space Biosciences Division, NASA Ames Research Center, Moffett Field, CA 94035 USA; 11 Embry-Riddle Aeronautical University, Department of Human Factors and Behavioral Neurobiology, Daytona Beach, FL 32114 USA; 12 Department of Botany, University of Wisconsin-Madison, Madison, WI 53706 USA; 13 Department of Biomedical and Health Informatics, The Children’s Hospital of Philadelphia, Philadelphia, PA 19104 USA; 14 Department of Pediatrics, Perelman School of Medicine, University of Pennsylvania, Philadelphia, PA 19104 USA; 15 Institute of Medical Genetics and Applied Genomics, University of Tübingen, Tübingen, 72076 Germany; 16 NGS Competence Center Tübingen (NCCT), University of Tübingen, Tübingen, 72076 Germany; 17 yuri GmbH, Meckenbeuren, 88074 Germany; 18 Ohio Musculoskeletal and Neurological Institute (OMNI), Heritage College of Osteopathic Medicine, Ohio University, Athens, OH 45701 USA; 19 The Feil Family Brain and Mind Research Institute, Weill Cornell Medicine, New York, NY 10065 USA; 20 SciLifeLab, KTH Royal Institute of Technology, Stockholm, 17165 Sweden; 21 Present Address: School of Chemistry, University of Glasgow, Glasgow, G12 8QQ UK

**Keywords:** Gene expression, Medical genomics, Molecular medicine

## Abstract

Future multi-year crewed planetary missions will motivate advances in aerospace nutrition and telehealth. On Earth, the Human Cell Atlas project aims to spatially map all cell types in the human body. Here, we propose that a parallel Human Cell Space Atlas could serve as an openly available, global resource for space life science research. As humanity becomes increasingly spacefaring, high-resolution omics on orbit could permit an advent of precision spaceflight healthcare. Alongside the scientific potential, we consider the complex ethical, cultural, and legal challenges intrinsic to the human space omics discipline, and how philosophical frameworks may benefit from international perspectives.

## Abstract (web summary)

High-resolution omics data have facilitated the ongoing Human Cell Atlas project. In this Perspective, Rutter and colleagues propose that a parallel Human Cell Space Atlas initiative would provide a platform for spaceflight-associated research and healthcare.

## Introduction

Humanity may be on the brink of establishing a new era of interplanetary space exploration that would witness crewed missions beyond low-Earth orbit (LEO) and a growing commercial spaceflight sector that would prompt a wider health range of individuals entering space compared to the selective cohorts of distinctively fit professional astronauts from previous generations. Numerous space organizations across the globe openly aspire toward landing humans on Mars in the coming decades, underscoring an overall international interest in what may be the next stage of human space exploration. Prominent examples include the SpaceX Mars program striving to initiate the first crewed Mars spaceflights in the late 2020s; the state-owned China Academy of Launch Vehicle Technology announcing the country’s goals to place humans on Mars in 2033; NASA (National Aeronautics and Space Administration, the United States space agency) issuing the Authorization Act of 2017, which declares objectives to send humanity to Mars in the early 2030s; Roscosmos (the Russian space agency) publishing aims to send humans to Mars in the early 2040s; and the United Arab Emirates publishing the Mars 2117 Project, which outlines a one-hundred-year plan to construct habitable communities for humans on Mars. Thus, there is a motive for scientists across the world to study how the human body responds to spaceflight and to develop countermeasures that improve the health and safety of crewed interplanetary missions.

**Fig. 1:**
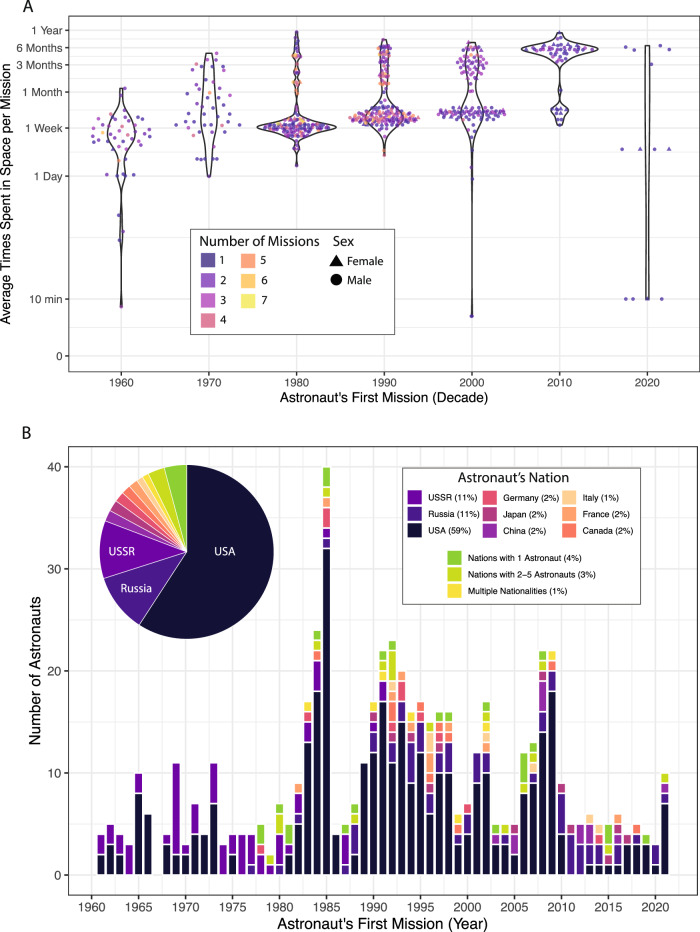
Space Missions. **A** Violin plots showing the average time a given astronaut spends in space per mission (calculated as total time in space divided by number of missions) compared to the decade the astronaut first went into space. Astronauts are colored by the number of missions they have been on, and shapes represent astronaut sex (females are triangles and males are circles). The average time spent in space ranged from minutes to one month in the 1960s, and from one day to under six months in the 1970s. In the 1980s through 2000s, the majority of astronauts spent an average of between one week and one month in space per mission, but many astronauts spent more than three months in space. Subsequently, in the 2010s, the majority of astronauts spent an average of over three months in space per mission, whereas in the early 2020 s, there was the widest distribution of average time in space, ranging from ten minutes to six months. **B** The number of astronauts who have been in space by nationality. Bar plot shows the number of astronauts by the year of their first mission whereas the pie chart shows the percentage of each nation’s contribution. Nations with only one astronaut to ever go to space are colored green (4%), nations with only between two and five astronauts to go to space are colored lime green (3%), and astronauts with multiple nationalities are colored yellow (1%). Data was scraped from supercluster.com on September 20th, 2021. Only astronauts who spent time in space and crossed the Kármán line are displayed.

Since the inception of human spaceflight, the duration that astronauts spend in space each mission has increased over time (Fig. 1A). Despite this, to date, only eleven individuals have resided in space for more than 300 consecutive days. Pioneering crewed missions to Mars would see humans embarking through space for even longer consecutive periods of time and in radiation environments for which there is limited knowledge about the impact on human physiology, warranting improvements in the autonomous space telehealth field. The risk of an emergency medical occurrence during space missions has previously been estimated at approximately 0.06 per person-year, which roughly equates to one event every 2.4 years for a crew of seven^1^. Multiyear planetary missions would prevent resupply and medical evacuation options and hence would require fully autonomous telehealth and triage protocols.

Various applications of artificial intelligence and molecular omics could likely advance the spaceflight telehealth field. Omics technologies allow for the quantification of large pools of biomolecules that influence the integrity and function of biology. Exploratory and untargeted omics techniques can measure analytes that are not predetermined. These studies can identify patterns of variance, which can generate new hypotheses. Additionally, integrating omics with metadata, including environmental and phenotypic measures, enhances the ability to establish robust links between environmental influences, omic variations, and phenotypic outcomes^2^. The NASA Twins Study integrated various omics platforms, including transcriptomics, epigenomics, metabolomics, and metagenomics, and highlighted omics as a potential biomedical research platform that may one day translate into the development of precision spaceflight healthcare^3^. The ambitious project discovered more than 8600 differentially expressed genes (DEGs) between an astronaut who resided for almost one year on the International Space Station (ISS) and their identical twin who resided on Earth; it is possible that any permutation of the DEG list could uncover biochemical pathways that hold keys to the development of therapeutic supplements and lifestyle recommendations that better protect health in space^3,4^. In this paper, we will consider how the dawn of diversified human exploration of deeper space may benefit from sophisticated advances in spaceflight nutrition and health that may be met in part with the addition of routine standardized omics.

Space omics efforts are now underway in multiple regions. In Japan, the “Living in Space” Grant-in-Aid for Scientific Research (KAKENHI) program uncovers biological responses to the space environment at various levels, ranging from environmental microbiomes to stress responses in humans, using omics technologies. In Europe, the Space Omics Topical Team (TT) supports and generates omics approaches to space biology^5^, and there are visible efforts to promote further development of space omics research among ESA and ESA state members^6^. In the United States, examples of space omics-related campaigns include the Precision Health Initiative and Systems Biology Translational Project through the NASA Human Research Program (HRP) and the Complement of Integrated Protocols for Human Exploration Research (CIPHER) project (“Human Research Program Update”, WH Paloski, Committee on Biological and Physical Sciences in Space (CBPSS) Virtual Fall Committee Meeting). The NASA GeneLab initiative provides an open access, collaborative analysis platform for space omics data collected across the world and unites international efforts through analysis working groups^7^. Canada operates terrestrial platforms to analyze human omics linked with biomedical data^8^, and the Canadian Space Agency (CSA) has announced plans for national space omics research, in addition to already participating in international space omics projects. China and Russia are also publishing human space omics research^9,10^.

Humans in spaceflight historically represented nations like the United Socialist Soviet Republic and the United States to a large degree (Fig. 1B). After the first couple of decades of human spaceflight, nations like Canada, China, France, Germany, Italy, Japan, and Russia have also been represented to a moderate degree (Fig. 1B). Individuals who have entered space now represent, even if in smaller numbers, more than thirty other nations: Afghanistan, Antigua and Barbuda, Australia, Austria, Belgium, Brazil, Bulgaria, Cuba, Czechoslovakia, Denmark, Egypt, Hungary, India, Iran, Israel, Kazakhstan, Malaysia, Mexico, Mongolia, Netherlands, Pakistan, Poland, Portugal, Romania, Saudi Arabia, Slovakia, South Africa, South Korea, Spain, Sweden, Switzerland, Syria, Ukraine, United Arab Emirates, United Kingdom, and Vietnam.

The Artemis program plans to soon reestablish a human presence on the Moon, for the first time in five decades, and construct a permanent lunar base to facilitate the future of human missions to Mars. Led by NASA and partner agencies JAXA, ESA, and CSA, the Artemis program includes signatories from about thirty countries and territories, reflecting on the theme of a more global human presence in space. Indeed, crewed missions to Mars would motivate philosophical thinking about humanity’s place in the universe, common to all humans. Many people believe such missions will only be enabled by worldwide collaboration due to the involvement of multiple countries and international organizations, the implications for many people across Earth, the sheer engineering demands, and the increasing global representation of space explorers^11^. Upcoming planetary missions will likely be multinational efforts, underscoring the need for global collaboration in regard to the science, culture, and ethics behind space exploration. A new age of human space omics may likewise require international input due to both the various cultural aspects and the added technological, ethical, and philosophical complexities of working with human subjects.

International Standards for Space Omics Processing (ISSOP) represents an international consortium of space omics scientists who formed after recognizing the need for standard guidelines in the emerging spaceflight omics discipline. ISSOP includes scientists with expertise across the full range of omics approaches who receive funding from academia, industry, and government agencies across a wide array of regions, including Japan, India, the Middle East, Canada, Europe, Russia, and the United States. By promoting communication exchange in the space omics discipline, ISSOP may be uniquely positioned to support the development of an informed framework early on that can help maximize scientific discovery and minimize ethical problems for an upcoming era of human space omics.

In this paper, we consider how a future of precision space healthcare could improve the safety of human health during long-term spaceflight. We then discuss how careful standardization of space omics data is one component that may help implement this goal. To this end, we propose the development of a human cell atlas under spaceflight environmental conditions that could assist as an openly available, global resource for foundational space life science research. We then consider the complex ethical, cultural, and legal challenges intrinsic to the general discipline of human space omics, and how philosophical frameworks would likely need to be established with perspectives from international ethicists.

### Precision healthcare in space

In the coming years, the spaceflight field will likely observe diversification of the flying population, extended mission durations, and exposure to harsher radiation beyond LEO. These factors motivate an upgraded space healthcare model where nutritional, supplemental, and pharmaceutical decisions could be tailored to multiple characteristics^12,13^. On Earth, there are already applications of precision health, where healthcare is informed based on omics, environmental, and lifestyle factors^14^. It seems probable that as connections between omics and spaceflight health ailments are slowly elucidated, similar approaches could be implemented in space. The purpose of customizing spaceflight healthcare would not be to reduce spaceflight participation, but to promote the health of increasingly diverse participation that better reflects the sustained presence of the full inclusive range of humanity in space one day^13^.

Countermeasure protocols for space missions can consist of evidence-based nutritional supplements, exercise regimes, stress-relieving techniques, and pharmacological interventions^15^. Moving forward, crew profiles could potentially improve medical risk assessment and countermeasures; optimize medical and nutritional payloads; increase crew safety and efficiency; and maximize the likelihood of mission success. In the next section, we will explore examples of how astronaut health and performance can be impacted by omic differences related to the metabolism of drugs, micronutrients, and macronutrients. We focus on metabolism because changes in mitochondria, the key hub of metabolism, are a fundamental biological feature of spaceflight^16^.

We note that the provocative nature of the spaceflight environment may induce a large number of physiological and molecular changes on rapid time scales that may not necessarily be of health-related importance. As a result, scientists must cautiously avoid overassociating spaceflight omics changes with clinical meanings. Indeed, most human gene association studies on Earth are associative with many genome-wide association studies (GWAS) providing initial results that failed to hold up upon further testing. The risk of overinterpretation is particularly relevant in the field of human space omics given the small sample sizes and the small number of studies^3^; the difficulty of securing suitable ground controls, both in omics and environment^3,17^; and the long number of years required to replicate findings. These problems are further amplified when studying the long-term effects of spaceflight outside of LEO. We emphasize that the following section mostly presents early evidence and even contradictions due to the current limitations of the human space omics field, and that much more work would be needed in the coming decades to assess the validity of the below preliminary findings and interpretations.

### Drug metabolism in space

Medical kits onboard the ISS contain pharmaceuticals to cover various medical events and emergencies, including injuries, illnesses, infections, sleep disruptions, motion sickness, and cognitive and behavioral health conditions^18^. Documentation of medication usage has not been consistent, but studies suggest about 94% of crew members used medications at least once during space shuttle missions^19^. Despite the common use of medications to manage health concerns on orbit, few studies have explored how to refine pharmaceutical applications in the unique environment of space. Preliminary evidence suggests that spaceflight may introduce impurity products, alter the physical appearance, and quicken the degradation of certain drugs before their expiration dates^20,21^. Although these investigations have been severely limited without adequate ground controls, they have underlined the need to verify if any spaceflight factors may affect pharmacokinetic and pharmacodynamic parameters that determine the pharmaceutical safety and efficacy, and the mechanisms responsible for these effects^22,23^. These factors might include the space environment itself (such as chronic low-dose radiation) and/or confounding extraneous factors (such as temperature, humidity, and dosage repackaging commonly used to meet the limited volume constraints on space vehicles)^18,22,24^.

Thorough metadata tabulation could eventually help unravel not only how to preserve drugs in space, but also how to better understand precision responses to such drugs. A recent pharmacogenetics study by the ESA demonstrated that allelic variation may influence the safety and effectiveness of how individual crew members metabolize drugs on the ISS. Researchers examined the 78 standard drugs permanently available on the ISS and found that the metabolism of 24 of them was significantly affected by individual variants in genetic polymorphism enzymes^25^. This discovery suggested that almost one-third of drugs on the ISS may warrant personal dose adjustments or alternative therapies for crew members who have allelic predispositions that can render them anywhere from poor metabolizers (with decreased drug clearance, increased plasma drug levels, and potential adverse drug response) to ultra-rapid metabolizers (with increased drug clearance, decreased plasma drug levels, and potential ineffective drug response). Populations throughout the world can have different frequencies of genetic polymorphism enzymes that affect drug metabolisms^26^; thus, pharmacogenetic screening of consenting astronauts from diverse backgrounds could better ensure equal representation^27^.

Personal astronaut drug-metabolism profiles could be generated based on the two reaction phases of drug biotransformation. The first drug biotransformation reaction produces a more water-soluble and less active metabolite usually through the hydroxylating enzyme superfamily known as Cytochrome P450 (CYP450). This enzyme superfamily is believed to account for 75% of total drug metabolism^28^. Indeed, CYPs have recently been suggested to be altered in flight due to alterations in insulin and estrogen signaling^29^. Several CYP450 genes are highly polymorphic, producing enzyme variants that cause variability in drug-metabolizing effects between groups. During mission planning, CYP450 genetic variant profiles for consenting astronauts could be generated and cross-referenced with mission drug lists to prevent scenarios in which crew members would otherwise metabolize drugs in harmful or inefficient ways^13^. The second drug biotransformation is usually a conjugation reaction wherein a small molecule binds to the drug metabolite and increases its solubility for excretion. For this situation, consenting astronauts could assess their pre-mission status of all nutrient cofactors and conjugation agents (such as glutathione, glycine, cysteine, arginine, and taurine) to inform their optimum conditions^13^.

One concrete example of how CYP allelic variants could inform treatments during space exploration relates to acute radiation sickness (ARS)^30^. ARS is an accepted risk on orbit, and common medications to treat its primary symptoms of nausea and vomiting include ondansetron and granisetron, which are both metabolized differently in the liver based on individual CYP genetic polymorphisms. Specifically, the CYP2D6 enzyme metabolizes ondansetron, and, hence, groups who are ultra-rapid metabolizers of the CYP2D6 pathway have a higher risk of still vomiting within one day of radiotherapy with ondansetron^31^. These groups may benefit from treating ARS with granisetron, which is instead metabolized by the CYP3A enzyme^31^. Another example of how CYP allelic variants could inform spaceflight therapies based on personal predispositions relates to sleep drugs, which are commonly used by crew. In fact, 78% of shuttle crew reported taking zolpidem and zaleplon, powerful sleep pills, for more than half of the nights of their missions^32^. CYP3A polymorphisms are known to affect zolpidem metabolism^33^, but not zaleplon metabolism^25^, and astronauts could use this knowledge to more safely tailor their sleep-related treatments.

It may also be meaningful to investigate optimal doses of medications that are more tailored to the demanding lifestyles of astronauts. For example, unlike many individuals on Earth, astronauts on the ISS are occasionally awakened by alarms during scheduled sleeping shifts in order to perform emergency tasks that require effective cognitive and psychomotor capabilities^34^. A study at NASA Johnson Space Center found that subjects who consumed a higher dose of zolpidem before sleep experienced significantly reduced cognitive and psychomotor performance during emergency awakenings; however, they found no similar impairments in subjects who consumed the lower dose of zolpidem, the dose of zaleplon, or the placebo before sleep onset^34^. Hence, even though the approved doses of zolpidem and zaleplon may be fit for most terrestrial applications, the unique duties during spaceflight may warrant adjusted recommended dosages of these, and possibly other, approved medications.

Even the metabolism of medications may be altered in spaceflight compared to on Earth. The kidneys, which play a major role in drug excretion, may reduce urine output during weightlessness^35^. The liver, which is the main organ that metabolizes drugs and xenobiotics, may also behave differently between terrestrial and spaceflight conditions, although results have been conflicting: While some studies have reported an increase in hepatic blood flow and size during spaceflight^36^, other studies have suggested a decrease in hepatic metabolism in space, which may roughly correspond to a decrease in hepatic blood flow due to the hypovolemia that occurs on orbit^37^. Clear elucidation of potential differences between drug metabolism in terrestrial versus orbital conditions will require further investigation, and any reliable findings could eventually be integrated for improved aerospace healthcare recommendations^27^.

### Micronutrient metabolism in space

Recent studies are elucidating tentative relationships between omics and micronutrient intake that may link to adverse health events in space. One key example of this is one-carbon metabolism, which involves the transfer of methyl groups from donors (such as folate, B12, choline, and betaine). We note that many of the donors are essential inputs that must be obtained from the diet. The enzymes that regulate one-carbon metabolism are produced from highly polymorphic methyltransferase genes; any possible ramifications for human spaceflight are only recently unfolding^13^.

As an example, spaceflight-associated neuro-ocular syndrome (SANS) is a unique and distinctive clinical manifestation. It includes optic disk edema (swelling), choroidal folds, and focal areas of ischemic retina (cotton wool spots). This disease is believed to present in over 20% of astronauts both during and after short and long duration spaceflight^38^. With no known terrestrial analogue, evidence-based countermeasures are only recently emerging; these include lower body negative pressure and nutritional supplementation^39^, the latter of which could be further refined through omics studies. Indeed, one research group examined 49 astronauts and discovered that common variations in one-carbon metabolism genes, combined with lower levels of vitamins B2, B6, and B9, appeared to be associated with SANS^40,41^.

In addition to poor neuro-ocular health, disordered one-carbon metabolism may play a role in bone fragility (with increased osteoclast activation and decreased osteoblast activity)^13^, hypertension (with increased intraocular pressure)^42^, and chromosome instability (with concurring folate deficiency increasing defective DNA repair)^43^. It is indicative that this single metabolic property may have wide-ranging impacts on some of the more perplexing health complications known to occur during spaceflight^13^. For these reasons, one-carbon metabolism is an example candidate for deeper research into a future precision space medicine approach.

There is likewise preliminary evidence that prolonged radiation exposure may interact with genetic polymorphisms that alter micronutrient metabolism, predisposing to disease in space. For example, there is precursory evidence suggesting that the space environment partially contributes to altered iron metabolism in astronauts^44^. Iron overload is believed to occur more often in individuals with allelic variants for hemochromatosis (HFE)^45^. Simultaneously, urinary magnesium (Mg) levels are reported to decrease during space missions, with a slight majority of post-spaceflight astronauts presenting with levels below minimum clinical guidelines^46^. Taken together, these provisional observations suggest that astronauts with HFE allelic variants may develop unusually high levels of iron when exposed to the space environment, which may induce oxidative stress and unstable DNA^47^. Given that Mg repairs DNA damage^48^, further research may be justified to examine whether convergent Mg deficiencies could complete a dangerous aggregation of risk events in a subset of astronauts who are susceptible to this array of environment, omics, and dietary intake parameters^13^. DNA stability is a leading human safety concern in space and hence it seems meaningful to investigate whether consenting individuals can benefit from optimizing any essential dietary inputs with relevance to DNA repair before, during, and after spaceflight based on precision risk profiles.

The supplementation of Vitamin D, another important micronutrient, is regularly integrated into spaceflight nutrition programs^49^. While Vitamin D is well-known for its influence on bone production, it impacts several more biological processes, including immune system modulation. Its effect on immunity seems to be mediated by the Vitamin D receptor (VDR), expressed by antigen-presenting cells and activated T cells^50^. Conversely, Vitamin D and VDR are necessary to maintain a healthy number of regulatory T cells^51^. Allelic variants of the *VDR* gene appear to be associated with better response to Vitamin D supplementation^52^, and allelic variants of genes (such as *7-Dehydrocholesterol reductase* (*DHCR7*), a gene related to sterol metabolism) are believed to be related to improved Vitamin D metabolism and insulin resistance^53^. Therefore, poor Vitamin D status and metabolism of individuals during space missions, which already have inherent stressful elements, might negatively affect the immune systems of astronauts, and this could potentially be mitigated through precision omics profiles.

### Macronutrient metabolism in space

Differences in macronutrient consumption needs have been linked to variants in genes, such as the *Retinoic acid receptor beta* (*RARB*) gene, the *DNA damage-regulated autophagy modulator 1* (*DRAM1*) gene, and the *Fat mass and obesity-associated* (*FTO*) gene^54,55^. Altogether, these variants appear to be associated with body composition, fat distribution, and obesity risk in relation to carbohydrate, lipid, and protein intake^54,55^. Initial studies have suggested that endocrine changes linked to spaceflight modify metabolism and strengthen its association with alterations in astronaut body composition and nutritional intake needs^56^. It may be possible that long-duration space voyages could exacerbate dietary deficits. The nutritional condition of astronauts appears to be affected by metabolic stress, changing gut flora, altered feeding behavior, vitamin insufficiency, and electrolyte imbalance^57^. Maintaining energy balance in space missions will likely be critical for maintaining body fat muscle homeostasis^58^.

These early findings imply a possibility that prolonged space exposure and dietary deficits may interact with genetic polymorphisms in a subset of astronauts to promote unusual pathologies that could be prevented by better nutritional information and health plans based on thorough research in the spaceflight omics discipline. It may hence be valuable to properly investigate and characterize any relationships between micronutrients, macronutrients, pharmaceuticals, omics, and various spaceflight environmental factors. These relationships are complex and underline the critical usefulness for rigorous standardization of space omics data and metadata so that meta-analyses may one day disentangle more confidently which factors are linked to preventable diseases at both the group and individual levels during spaceflight.

### Standardized space omics collection

Standardization of space omics data and metadata would be an important component of a successful human space omics discipline. JAXA is currently conducting human liquid biopsy studies on the ISS. The main purpose of the studies is to investigate how the space environment affects humans at the tissue level. Besides fat and skin samples, which can be risky, whole-body solid tissue biopsies cannot typically be conducted in humans in space. As a result, the project proposes to perform minimally invasive liquid biopsies in astronauts. Liquid biopsies can detect cell-free components (microRNA, RNA, DNA, and extracellular vesicles) in the blood and scientists can estimate which tissues the cell components are derived from, allowing for full-body monitoring of omics responses. While solid biopsies only reflect a single time point of a single site on a single tissue, liquid biopsies can allow for a less invasive assessment of extracellular DNA and RNA in the plasma, which can represent molecular responses of the internal tissues in the body and can be obtained at repeated timepoints. The project proposes to collect astronaut blood samples at multiple time points before, during, and after living on the ISS. Murine and human plasma samples may be processed using the same processing protocol, thereby allowing for direct comparison of human and veterinary patients in a minimally invasive fashion for both species. These unique data collections are expected to enable cross-species integrative data analysis of space environment effects on mammals in a manner that is less technically and ethically complex than previous methods.

Moving forward, the majority of sampling on astronauts would likely consist of liquid biopsy approaches and other non-invasive and minimally invasive procedures. Traditional tissue biopsies carry a risk of infection and localized pain which could compromise astronaut health and performance in physical tasks such as spacewalks. Furthermore, less invasive sampling methods are often quick to perform and do not require significant recovery periods between samples, which can allow for more frequent sampling and thus greater temporal resolution of omic changes throughout missions. The feasibility of orbital sampling and readout can be explored for various biological sources, such as saliva, sweat, tears, urine, and nasal discharge, some of which have been reviewed in the context of human spaceflight elsewhere^59^. An increase in capabilities for inflight omic processing and readout could also enable inflight interventions to maintain health, such as nutritional supplements based on gene regulatory changes related to metabolism of specific vitamins.

New metadata normalization is another example of a rising standardization challenge that comes with the arrival of human space omics projects. Some key metadata parameters that should be collected from humans include environment, diet, nutrition, psychosocial dynamics, lifestyle, medical history, anthropometrics, and phenotypes^13^. Many of these metadata parameters have not been necessarily collected in space and hence have no standardization methods. For instance, wearable technology could be implemented to provide metadata of astronaut health, similar to fitness trackers that have been successfully used on Earth to measure various medical parameters, including increased insulin resistance, which has also been observed in spaceflight^60,61^. At the same time, metadata can be misused to identify individual astronauts, an ethical problem that, unlike in model organism studies, has to be addressed. With a mission to continuously improve recommended metadata normalization protocols for space omics data, ISSOP may help spark discussions needed to resolve some of these challenges.

In addition to best efforts to standardize space omics studies^62^, increased resolution may help interpret allelic variants associated with disease risks by more precisely pinpointing cell types and states involved in biology. Achieving this goal relates back to our project exploring an example set of alleles that are putatively linked to protective mechanisms, and their implications for therapeutic compound discovery and improved spaceflight nutrition and lifestyle recommendations^63^. As described next, extending standardized approaches toward space omics data that is higher in resolution could enable the generation of cell space atlases, which could further push forward a developing field of precision space healthcare.

### Human cell space atlas

To date, bulk transcriptomic technologies, such as RNA-sequencing (RNA-seq) and microarrays, have mostly been used to understand how spaceflight impacts physiology. These approaches have characterized physiology at the tissue level, composed of billions of diverse cells, rather than at the individual cell level. However, to obtain a thorough understanding of the complex dynamics that spaceflight causes in humans, there is a need to increase the analysis resolution level. In the Space Omics and Medical Atlas (SOMA), researchers recently collected high-resolution omics data from commercial astronauts during the Inspiration4 mission; the project included multi-omics spatial mapping, single-nucleus RNA-seq (snRNA-seq), single-cell RNA-seq (scRNA-seq), and single-cell Assay for Transposase-Accessible Chromatin sequencing (scATAC-seq). Civilian missions may greatly contribute to advancing the spaceflight omics field, given that many commercial spaceflight participants have expressed voluntary interest in participating in space biomedical research and sharing data for biobanks and scientific publication^64,65^. These technological advances have created an unprecedented opportunity for in-depth molecular studies in space biology in the short- and middle-term future.

In recent years, researchers on Earth have used high-resolution omics to create a plant cell atlas (PCA)^66^, mouse cell atlas (MCA)^67^, and human cell atlas (HCA)^68^. These initiatives aim to construct thorough spatial maps of all cells in organisms as references for research, diagnosis, monitoring, and treatment of disease^69^, and assist in the development of better therapies. Contemporary routine blood tests that provide rough counts of white and red blood cells could become acutely more informative if cell types and states can be discerned with finer granularity. This could lead to improved diagnostic tests that detect infections *before* clinical symptoms are present, representing a shift from reactionary treatment of health issues towards a preventative approach^69^.

**Fig. 2:**
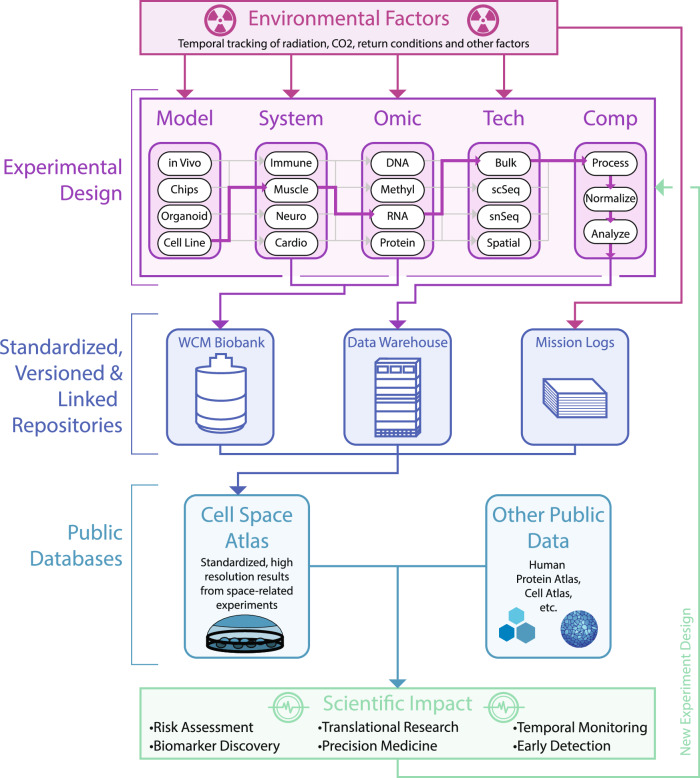
Cell space atlas. Multi-omic experiments, whether on Earth or in space, have a number of complexities when designing and comparing results to other published work. Namely, there are numerous models which could be leveraged to investigate the molecular (omic) changes in different organ systems using different technologies, which can then be processed and analyzed in numerous ways. Further, experiments conducted in space may be more influenced by environmental factors that are either regulated within the craft (such as oxygen) or not (such as radiation). These environmental factors are crucial to understanding results and can drastically vary by experiment. Given these complexities, understanding the environmental factors during a mission and the exact experimental design (including acquiring and analyzing the data), and standardizing them across agencies will be crucial to the development of aerospace multi omic analyses. Further, given the overall cost of these experiments as well as the limited resources to conduct them, this centralized and normalized database, which is accessible to other scientists, can assist our understanding of spaceflight risks, their counter measurements, and monitoring.

As society becomes increasingly spacefaring, one can envision the development of parallel initiatives to the PCA, MCA, and HCA that are specific to the space environment (Fig. 2). A plant, mouse, and human cell space atlas (PCSA, MCSA, and HCSA) could improve our understanding of how each system responds to spaceflight and hence enhance health monitoring capabilities during prolonged space missions. Having access to the different changes occurring at the spatial cell type level in key organs between ground control and flight conditions would allow researchers to better understand how cell-cell interactions are altered by spaceflight^70,71,72^.

These insights could therefore lead to a more detailed comprehension of how various botanical organisms, veterinarian patients, and human patients respond to spaceflight in order to design new and substantially more precise treatments as countermeasures for health in space. The cell space atlases could serve as openly available international resources developed at the start of the human space omics era that span multiple generations to come, not only to characterize cellular responses to spaceflight but also to investigate how cell cross-talk is altered when the system is exposed to extreme environmental conditions like space.

Human and model organism cell space atlases could be constructed in a minimally invasive manner, using in vitro and ex vivo technologies. For example, tissue chips (organs on chips) could be viable options for high-resolution space omics studies^73^. In the past several years, a series of experiments have tested tissue chips on the ISS through the Tissue Chips in Space initiative, which is a partnership between the ISS National Lab and the National Center for Advancing Translational Sciences (NCATS) at the National Institutes for Health (NIH)^74^. Tissue chips are composed of human cells grown on artificial 3D scaffolds to model the structure and function of human tissues, allowing researchers to assess how major organs and systems in the human body respond to the extreme environment of space. Real human tissues can also be studied in spaceflight following protocols similar to the Suture in Space initiative, where living tissue from biopsies are extracted and sutured together to serve as models to better understand physiological mechanisms in space, including wound repair and regeneration^27^. Engineers have already developed miniaturized equipment and automation procedures for tissue chips to be employed in low capacity during flight missions. ESA plans to develop a 3D bioprinter for the ISS that could generate human tissue constructs in microgravity to study the complexity of cellular component responses to the space environment^27^. In addition to their role in potentially developing cell space atlases, personalized chips and tissues could also be exposed to extreme environments before missions to assess individual risks, and then could be placed on spaceflight to monitor health changes and test prospective countermeasures.

We note that the omics methods used to construct cell space atlases may depend on whether sample processing occurs during spaceflight and/or back in terrestrial labs. For example, scRNA-seq approaches require fresh material, whereas snRNA-seq approaches can use frozen samples^75^. Spaceflight restrictions may also limit omics approaches: For instance, methods may initially be proteomics-based on orbit to successfully accommodate compact equipment that can operate at low power^13^. This could potentially lead to the generation of a human protein space atlas that integrates spatial mapping of human proteomics into atlas efforts, paralleling the Human Protein Atlas project already initiated in terrestrial healthcare^76^.

Overcoming these technical boundaries and optimizing the quality of these technically novel datasets would require input from experienced omics scientists with extensive knowledge about spaceflight biology. ISSOP is composed of members with stated missions to routinely update recommended sample processing guidelines for space omics datasets to allow for better harmonization of data and increased gain of knowledge^77^. Members have expertise related to the processing of multi-omics data samples, and several ISSOP members are prime contributors to the previously mentioned JAXA and NASA projects utilizing cutting-edge and high-resolution omics techniques. As a result, ISSOP could serve as a community that is integral for propelling the space omics field into the next stage of innovation with projects related to themes such as the construction of cell space atlases.

Alongside the technical challenges of constructing a human cell atlas for spaceflight, the generation and usage of human space omics data poses considerable ethical, cultural, and legal challenges that would need to be carefully resolved through policy development. Indeed, one motivation of the current perspective paper is to provide examples of the upcoming ethical and legal complexities inherent to the nascent human space omics discipline in order to encourage earlier discussions amongst international ethicists, philosophers, scientists, and the public community.

### Ethical and policy considerations

**Fig. 3:**
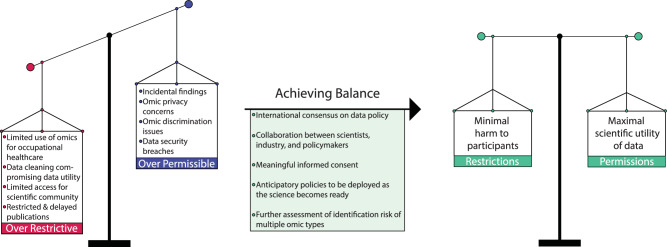
Ethics. Policies pertaining to the collection, storage, and usage of omics data from consenting astronauts and spaceflight participants would need to be carefully balanced. Thorough discussion amongst international ethicists could ensure that such policies are designed such that they are not so restrictive that they significantly limit the potential for scientific progress and improved occupational healthcare in space, and not so permissible that they expose participants to ethical harms.

As a consequence of the increasing adoption of human omics research in space, it has become crucial to increase standardization of policies for regulating the collection, storage, access, and usage of astronauts’ (sometimes called spaceflight participants, or SFPs) omics data. Based on recommendations from a 2014 report by the National Academy of Medicine (formerly the Institute of Medicine)^78^, NASA instituted a policy (NPD 7170.1) regarding the collection and usage of genomic data for human research. However, we are not currently aware of other prominent space agencies - or commercial spaceflight companies - publishing public policies regarding astronaut or crew omics data. Notably, the United Nations recently formed a working group on “Space and Global Health”. In their draft resolution (A/AC.105/C.1/L.402), they encourage Member States to “establish a policy-enabled environment and governance mechanisms, with due consideration of legal and ethical issues, for removing barriers to the effective use of space-based technologies, including telemedicine solutions”. The current rarity of human omics collection and curation can be viewed as a barrier to the development and deployment of emerging space-based health technologies, including precision healthcare. Thus, in the following section, we consider legal and ethical challenges pertaining to policymaking in this context, concluding with some thoughts on how best to achieve balanced policies which support space omics research while protecting the rights of the participants (Fig. 3). Ethical considerations of human omics research is an emerging area for spaceflight^79,80,59^, this is just part of the bigger picture of space ethics^81^, and human omics research on Earth^82^.

### Dangers of restrictive policies: scientific potential unused

As mentioned throughout this manuscript, standardized collection and curation of human space omics data has great potential to accelerate scientific research into human health in space and enable the deployment of precision medicine technologies to improve occupational healthcare during spaceflight missions. Therefore, underutilization of the scientific and healthcare potential for human space omics data is the primary danger of overly restrictive policies.

Firstly, if policies restrict the operational usage of human space omics data in occupational health processes, such as countermeasure design and long-term health monitoring, the full potential for reducing health risks might not be realized. Consequently, this could endanger crew members, reduce likelihood of mission success, and potentially infringe upon the duty of care space agencies have for their astronaut employees. This duty of care includes not just the immediate health and safety of astronauts during missions, but also their long-term health due to occupational exposures such as cosmic radiation^78^.

Secondly, if policies do not allow for sufficient data to be collected and processed from humans in space for research purposes, the pace of space life sciences research will be slower, with increased dependency on ground-based analogs. Similarly, restrictive policies for data accessibility/sharing would also slow the pace of research. When investigators contribute data to the scientific community through open-science initiatives, it can be analyzed via varied approaches, including comparatively to other datasets to yield novel insights. In the context of spaceflight omics data, the value of open-science has recently been demonstrated through a large-scale multi-omics analysis comparing datasets from the NASA GeneLab biorepository, to uncover mitochondrial dysregulation as a key hub of the biological response to spaceflight^83^. Biorepositories can be leveraged with “Big data” approaches, where patterns such as radiation sensitivity, can be learned via the application of machine learning methods to collections of multi-omics datasets^84^. With increased collection of omics data from humans in space, these kinds of approaches could be applied to human spaceflight, such as to predict susceptibility to radiation-based health issues in individual crewmembers^85^. Limiting accessibility to human space omics data creates a barrier to analysis efforts, thus every decrement from complete open access reduces the scientific utility of the data.

A final consequence of restrictive policies is reductions in the scope and precision of the data provided to the wider scientific community. For the NASA Twins study, authors of one follow-up paper that accessed and reanalyzed the original study data indicated that single nucleotide polymorphisms (SNPs), indels, copy number variations (CNVs), and structural genomic elements could not be included in the publication for confidentiality purposes^86^, thus limiting the scope of their public facing analysis. Regarding precision, efforts to anonymize and sanitize the data prior to data sharing - including data aggregation approaches and withholding metadata - could reduce the scientific utility, particularly as individual differences, such as gene variants associated with susceptibility to health issues, are essential information for unlocking precision healthcare approaches^63^.

### Dangers of permissible policies: exposure to ethical harms

On the other hand, while scientific utilization of human space omics data could reduce health risks for crews, if policies are overly permissible, omics research could also lead to harm by exposing human subjects to ethical issues. In the case of space agencies, this would also infringe on their duty of care to their astronaut employees. Due to the familial nature of genomics, these issues may also affect family members. Notably, compared to typical terrestrial cohorts, many of these ethical challenges are amplified by the unique nature of the spacefaring population; astronauts are easily identifiable due to their current rarity and status as public figures^79^.

One ethical issue that is particularly amplified by the standing of astronauts is that of privacy. The term “genetic privacy” is used ubiquitously, yet privacy breaches could apply to any identifiable omic-based personal data. Privacy breaches would involve disclosure of this data against the will of the participants, which could lead to psychological harm. Specifically, policies may be considered overly permissible if they fail to account for the risks of identifiability from the full range of omic data types and data formats. It is well understood that individuals can be identified using genomics data, such as SNPs^87^. However, identification via other omic data types, such as transcriptomics^88^, proteomics^89,90^, microbiomics^91^, and combinations of omic types (multi-omics)^92^, is a developing area of the literature. Identifying specific participants becomes possible through “linkage attacks” when phenotypic information is available in addition to the omics data^88^. Due to the nature of astronauts as public figures, phenotypic information such as ethnicity, age, and biological sex is readily available to the public. The small population size also means that the number of possible matches is limited, increasing the likelihood of successful linkage attacks. Aside from linkage attacks, privacy may also be violated via attacks on the data storage system. Thus, policies may be considered overly permissible if they do not ensure appropriate security and safeguarding mechanisms against data breaches.

Relating to the issue of privacy is the ethical issue of genetic discrimination, which refers to individuals receiving differential or unfair treatment based on their genetic data. Policies permitting space agencies to use omics data predictive of health issues, such as genetic variants, in astronaut selection or during processes such as flight assignment, could be seen as discriminatory. Indeed, many countries worldwide have enacted laws to prevent the use of genetic information in employment decisions^93^. It is worth noting that this is somewhat of a controversial and complex topic; for example, in the United States, the military can use routine genetic screening results to inform assignment decisions, such as to withhold deploying troops with G6PD deficiency to locations which would require them to take antimalarial drugs, since doing so can cause life-threatening hemolytic reactions^94^. While the United States Space Force could present an interesting exception, NASA is not a branch of the military, so it is subject to the Genetic Information Nondiscrimination Act 2008 (GINA), and therefore it would seem that it cannot use omic information in assignment and employment decisions^79^, which is also aligned with the current NASA policy (NPD 7170.1). However, even with precision medicine approaches, sending an individual with a genetic predisposition to spaceflight-associated risks, such as radiation susceptibility, on a long-duration mission to Mars may still increase the likelihood of a serious medical incident. Thus, it has been suggested that omic information would be useful for spaceflight selection and assignment processes^95^. This presents an ethical dilemma, where on the one hand, using omic information for flight assignment without the individual’s consent could be seen as discriminatory, and on the other hand, not doing so could potentially endanger the crew and reduce likelihood of mission safety and success. An additional consideration here is that some spaceflight-relevant genetic mutations may be linked to certain populations on Earth, which raises further issues of equity^63^. Using omics for precision healthcare does appear to be supported under the current NASA policy and GINA^80^, and this could hopefully decrease health risks associated with individual differences during spaceflight missions to an acceptable level, while refraining from using omics information for employment selection and flight assignment. Altogether, this balance may align with what is perhaps the current consensus for an appropriate compromise, based on regulations in many regions worldwide.

One further ethical issue that will be faced by space agencies and commercial spaceflight companies collecting astronaut omics is the risk of incidental findings^80^. For example, collection and analysis of astronaut omics data could reveal unexpected findings, such as predisposition to late onset Alzheimer’s disease. In some cases, these findings may not be medically actionable. Disclosing these findings to the astronaut could cause them and their family members psychological harm. Policies would be seen as overly permissible if they do not factor in the risk of incidental findings and fail to implement appropriate and clear procedures for disclosure and genetic counseling.

### Keys to developing balanced policies

Ultimately, policymaking for human space omics should be considered as a balancing act between developing policies that are not so overly restrictive that they limit the scientific potential of the data, and not so overly permissible that they invoke risk and expose participants and their families to harm. Standardization of policies could ensure that an appropriate balance is struck, safeguarding against ethical risks, while enabling appropriate accessibility for scientific and healthcare utilization of the data. This need for balance is embedded into the mixed role of space agencies, as research institutions and clinical care providers for astronaut employees^80^. While each dataset may still need to be considered on a case-by-case basis, such as for assessing identifiability of the data, clear policy frameworks could reduce bias and help to prevent unwarranted delays associated with unstandardized policy. As the number of humans and enterprises in space increases, and the understanding of omics increases in the context of ethical issues including privacy and discrimination, standards will need to be continually reassessed and updated.

It is worth noting that while ethical challenges such as privacy are certainly amplified at present by the relatively unique standing of astronauts as a small cohort of figures in the public spotlight, to some extent, these issues are also shared by rare disease cohorts and elite athlete cohorts on Earth, and so existing policies in areas including handling incidental findings and data sharing appear to present an apt starting point for discussion^96^.

It is essential that standardization of policymaking for human space omics be considered with an international perspective, as space agencies and commercial companies are subject to different laws based on geographical region, and may also be influenced by cultural differences^81,97^. For example, laws surrounding genetic discrimination and handling of incidental findings vary internationally^93,98^. Where appropriate, policies should aim to find harmonious solutions within the framework of internationally established legislation, such as the Declaration of Helsinki^99^, respecting the rights of the human data subjects while giving careful consideration to the specific challenges of the astronaut or crew population and the societal role of human spaceflight. On the ISS, the IGA (intergovernmental agreement) extends the jurisdiction of countries to their registered elements and personnel; for example, European law applies to European astronauts and the Columbus laboratory module on the ISS, with the existence of member state national laws adding further complexity^59^. Conversely, the Human Research Multilateral Review Board (HRMRB) is a review board between international partners to ensure that all human subject research onboard the ISS is conducted ethically according to internationally agreed principles. It is likely that future missions, including commercial missions involving multi-national participants, will require similar navigation of international laws to create policies. This need to design harmonious international policy raises concerns over fairness; for example, if a country has more restrictive laws in areas such as data protection, it may become challenging for citizens of that country to participate in international space missions, omic studies, and omic-based technologies such as precision healthcare.

Similarly, given the role of international collaboration in human spaceflight research, establishing clear and harmonious policies for lawful sharing and processing of human space omics data will be crucial^100^. Where human space omics data is deemed to be identifiable, databases should adhere to high standards of security, and access levels or other technical solutions for reducing ethical harm risks should be considered^101^. For example, data sanitization methods can manipulate the data to reduce the risk of identification^88^, but should be balanced against the potential loss of scientific utility. Additionally, federated approaches^102^, such as federating learning for training AI models without moving the data across jurisdictions, could prove useful^103,104^. In adherence to FAIR (Findable, Accessible, Interoperable and Reusable) principles^105^, human space omics data should be as “as open as possible, as closed as necessary”, in order to maximize scientific utility while protecting the rights of the participants.

Furthermore, an important aspect of developing appropriate policies will be striving for meaningful informed consent from participants, both in terms of consenting to venture into space, and consenting to the collection, usage, and sharing of their omics data. To the former point, omics technologies, such as predicted risk based on individual differences, could help to provide participants with additional data, enabling greater decision autonomy to improve the informed consent process for space travel. Broad consent has been proposed as a potential solution to gain consent in the context of omics research, where it may be hard to define future uses of the data at a granular level, and the identifiability risks of different omics data types may be challenging to accurately quantify^106^. When carefully implemented, it has been argued that broad consent may be an appropriate choice for obtaining consent in the context of space omics studies and has been used in the recent commercial spaceflight health data repository established by the Cornell Aerospace Medicine Biobank and the Translational Research Institute for Space Health (TRISH)^65^.

Discussions regarding the development of appropriate policies need to commence early, so that anticipatory policies are ready as the science is ready^80^. With deep molecular profiling of astronauts now at the forefront of space biology research, and a key part of the climate of commercial spaceflight and ambitious missions outside of LEO, now would seem the opportune time to kick-start discussions into appropriate policy design. ISSOP, with its international membership and links to NASA GeneLab and the ESA Space Omics Topical Team, is well positioned to help guide standardization of policy-making regarding astronaut or commercial crews’ omics data, particularly from a scientific perspective. ISSOP can work closely with groups such as the Global Alliance for Genomic Health (GA4GH) to merge expertise in spaceflight omics research and terrestrial omics policymaking, which could help to ensure that policies maximize scientific utility while protecting the rights of the astronauts and crews.

## Conclusions

In the coming years, humans may venture into space for unprecedented durations and distances outside of the Earth’s radioprotective magnetosphere. Resupply and evacuation would be precluded in some cases, calling for a modernized spaceflight nutrition, wellness, and telehealth field that would likely incorporate molecular omics. Precision healthcare is becoming more commonplace for populations on Earth, where international consortiums are using high-resolution omics to construct the Human Cell Atlas. It seems probable that as increasingly large and diverse populations enter space, the purposeful implementation of routine standardized omics with consenting participants during spaceflight could similarly permit precision space healthcare and a Human Cell Space Atlas. The cell space atlas could serve as an open access, global reference for basic space life science research. While human space omics could catalyze critical improvement of safety measures for future space travelers, numerous ethical, cultural, and legal challenges would need to be carefully surmounted from global viewpoints. This manuscript provided examples of both the potential healthcare benefits and the ethical complexities that will likely be inherent to the human space omics discipline as a means to encourage anticipatory discussions amongst international ethicists, philosophers, scientists, and the public community.
